# Hanging Noncalculous Gallbladder

**DOI:** 10.1155/1996/60802

**Published:** 1996

**Authors:** E. Tzardinoglou, J. Prousalidis, S. Apostolidis, C. Katsohis, H. Aletras

**Affiliations:** A. Prop. Surg. Clinic Department of Medicine Aristotles University of Thessaloniki AHEPA Hospital Thessaloniki Greece

## Abstract

The removal of acalculous and not acutely inflamed gall-bladder in patients with typical biliary pain remains a questionable procedure. This study was conducted to present our experience. In the period 1982- 90, 1089 cases of calculous and acalculous gallbladder disease were treated in our clinic. In this period, 27 patients were subjected to cholecystectomy because of an acalculous, non inflamed gallbladder which was elongated lying in an abnormal position with a long cystic duct. The mean duration ofsymptoms supportive of cholelithiasis, was 5 years. Oral cholecystogram and ultrasonography led to the diagnosis and other causes ofchronic abdominal pain were excluded. There were 13 lumbar, 9 pelvic and 5 iliac gallbladders, with poor function in 20 of them. During cholecystectomy, the organ was invested by peritoneum and suspended in 7 cases from a mesentery. On pathological examination mild chronic inflammation was reported in 19 cases and minimal changes in 8. The minimum follow up was one year and the maximum 9 years. Complete relief of symptoms was achieved in all the cases. In conclusion, cholecystectomy should be offered in these symptomatic "hanging" gallbladders.


*HPB Surgery*, 1996, Vol. 9, pp.137–139
Reprints available directly from the publisher
Photocopying permitted by license only

© 1996 OPA (Overseas Publishers Association)
Amsterdam B.V. Published in The Netherlands
by Harwood Academic Publishers GmbH
Printed in Malaysia

### Hanging Noncalculous Gallbladder

E. TZARDINOGLOU, J. PROUSALIDIS, S. APOSTOLIDIS, C. KATSOHIS
and H. ALETRAS

A. Prop. Surg. Clinic, Department of Medicine, Aristotles University of Thessaloniki, AHEPA Hospital, Thessaloniki, Greece

*(Received 28 October 1993)*

The removal of acalculous and not acutely inflamed gall-bladder in patients with typical biliary pain remains a questionable procedure. This study was conducted to present our experience. In the period 1982–90, 1089 cases of calculous and acalculous gallbladder disease were treated in our clinic. In this period, 27 patients were subjected to cholecystectomy because of an acalculous, non inflamed gallbladder which was elongated lying in an abnormal position with a long cystic duct. The mean duration of symptoms supportive of cholelithiasis, was 5 years. Oral cholecystogram and ultrasonography led to the diagnosis and other causes of chronic abdominal pain were excluded. There were 13 lumbar, 9 pelvic and 5 iliac gallbladders, with poor function in 20 of them. During cholecystectomy, the organ was invested by peritoneum and suspended in 7 cases from a mesentery. On pathological examination mild chronic inflammation was reported in 19 cases and minimal changes in 8. The minimum follow up was one year and the maximum 9 years. Complete relief of symptoms was achieved in all the cases. In conclusion, cholecystectomy should be offered in these symptomatic "hanging" gallbladders.

Symptomatic patients with calculous gallbladder are readily subjected to cholecystectomy with good results. In contrast, the management of cases with acalculous, uniflammed gallbladder with biliary symptoms is puzzling and the results questionable[1].

The reason for this clinical problem is multi-factorial, but anatomic, functional, or pathological changes can be responsible[2,3], alone or in combination.

This study discusses the management of symptomatic "hanging" gallbladder, which we have found to be an infrequent but not rare anatomic anomaly.

### MATERIALS AND METHODS, RESULTS

In the A. Propedeutic Surgical Clinic, between 1982 and 1990, 1089 patients with calculous or acalcoulous gallbladder disease were managed. In this period 27 patients with chronic biliary pain attributed to a floating gallbladder in an abnormal position, were surgically treated. There were 6 men and 21 women. The age ranged from 31 to 74 years with an average of 56 years (Table I).

This history, physical examination and laboratory data were similar to that of cholelithiasis and chronic cholecystitis. The mean duration of symptoms was 5 years. Other abdominal diseases have been excluded through a systematic approach with upper GI series in all cases. Oral cholecystogram and ultrasonography, performed in all the cases, showed the abnormal shape and position and gave the indication for operative intervention (Figure I). IV cholangiography was performed in 15 and CT scan in 10 cases. The normally formed gallbladder has been found in the line of iliac crest (13 cases), major pelvis (9 cases) and the minor pelvis (5 cases) (Table II). Besides this positional ano-

**Table I** Sex and age distribution of patients with hanging gallbladders

| | *Patients* |
|---|---|
| Men | 6 |
| Women | 21 |
| Range of age | 31–74years |
| Mean age | 56years |

*Correspondence to:* Dr J. Prousalidis A' Prop. Surgical Clinic, AHEPA Hospital, Stil. Kiriakidi 1, 54636 Thessaloniki, Greece maly, all the gallbladders were elongated and 20 of them had malfunction on cholecystogram.

In all of the patients, cholecystectomy without operative death or postoperative complications was performed. Operative findings included a gallbladder which was circumferentially invested by peritoneum. In 7 cases, the organ was found to be suspended solely by the cystic artery and cystic duct. In 20 cases, the suspended gallbladder was found to have the cystic artery and duct embedded in a kind of mesentery. Intraoperative cholangiography, through the cystic duct, was performed in 10 cases and supported the diagnosis. On pathologic examination of the gallbladder, in 12 cases mild chronic cholecystitis and in 8 cases minimal inflammatory changes were reported.

Follow up examination, 1–8 years after surgery, revealed complete relief of postprandial pain in all cases. A mild degree of flatulence persists in 5 patients.

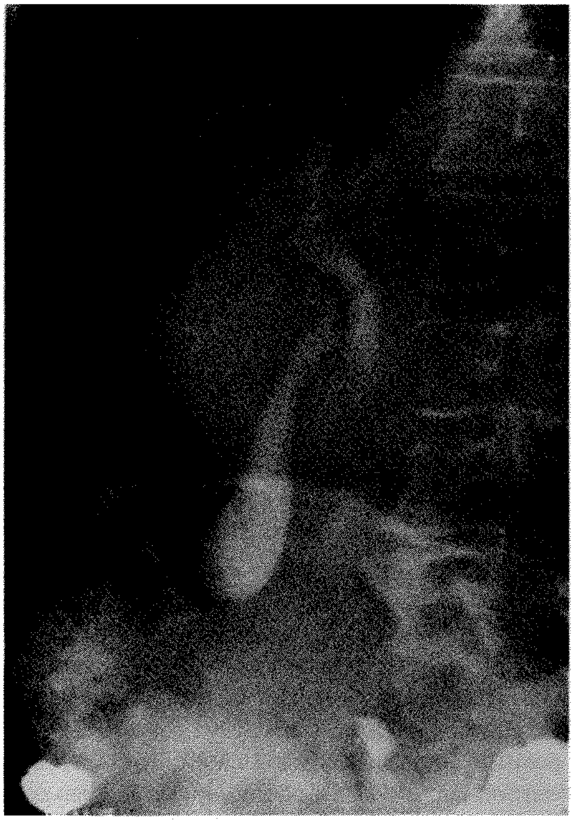

**Figure 1** Iliac gallbladder. (Note the oblong gallbladder lying in the minor pelvis)

**Table II** Operative findings in 27 cases with hanging gallbladders

| *Abnormal position* | *Patients* |
|---|---|
| Lumbar | 13 |
| Pelvic | 9 |
| Iliac | 5 |
| Total | 27 |

### DISCUSSION

The indication for elective surgical treatment of non-inflamed, acalculous but "symptomatic" gallbladder is sometimes mandatory[1]. The right upper quadrant pain, usually related to meals, is not severe but chronic and persistent and those afflicted become impatient and seek treatment[2].

Besides the other conditions associated with chronic acalculous gallbladder disease[3], it is our impression that an abnormal position of the gallbladder is sometimes responsible for similar symptoms. In our cases, the pearshaped organ, which normally is attached to the underface of the liver, was found to be oblong and "hanging" in a rather low position. In these patients the operative diagnosis is generally delayed due to necessary exclusion of other abdominal diseases [1,2,3]. Absence of other biliary, pancreatic or GI disease was documented with intravenous cholangiography, cystic duct cholangiography, sonography, CT scan, GI series and biochemical profiles. Papillary stenosis evaluated by some authors with endoscopic[4] or intraoperative[5] manometry and cholangiopancreatography[6] was, in our cases, excluded by meticulous assessment of the patient's history, preoperative or peroperative cholangiography and normal values of liver and pancreatic anzymes. During cholecystectomy we do not, like others[5], explore the bile duct or papilla in the absence of a dilated bile duct, bile duct stones, or abnormal bio-chemical profile.

One possible cause of symptoms in patients with hanging gallbladders may be the rotation of the organ. Incomplete (less than 180 degrees) or complete (more than 180 degrees) torsion of the floating gallbladder and adjacent cystic duct and mesentery has been reported[7,8]. The first type of torsion has a mild picture which disappears after spontaneous detorsion without any vascular impairment. In contrast, the second type of torsion produces a vascular compromise which results in gangrene and finally rupture of the organ. None of our patients required an emergency operation due to complete rotation of the gallbladder, but the repeated attacks of pain, in some of our patients, although not documented in the operating room, may have been due to reccurent episodes of incomplete rotation. We believe that the long spiral duct of the "hanging" gall-bladder is possibly the main reason for the malfunction and clinical symptoms. This is further supported by oral cholangiography which showed delayed emptying of contrast material from the gallbladder. The cure rate of patients with acalculous gallbladder disease after cholecystectomy varies in the literature from 65% to 100%[1,2,9,10]. The results of this study approached the level of complete relief (Table III). All of our patients had an anatomic problem of the gall bladder, which may not be as asymptomatic as has been described in the past[11], needed operative treatment. The patients with acalculous gallbladder disease reported in other series do not form a homogenous group but one with a different degree of severity of clinical, radiographic and pathological findings. This may explain the difference in the success rate.

**Table III** Results of Cholecystectomy in similar studies of acalculous gallbladder in the literature

| *Author* | *% cured* |
|---|---|
| Glen et al (1956) | 65 |
| Reid et al (1975) | 100 |
| Keddie et al (1976) | 90 |
| Gilliland et al (1990) | 75 |
| Present series (1990) | 100 |

We suggest that floating gallbladders without stones but with biliary colic, excluding other disease states, should be surgically treated. Besides the possible alleviation of symptoms in these cases, cholecystectomy could be also effective in the management of gallstones not seen on cholecystogram[9], or of small papillomas[2], which carry a malignant potential[12].

### REFERENCES

1. Glenn F. Mannic H., 1956. The acalculous gallbladder. *Ann. Surg.*; **144:** 670–680.
2. Keddie N.C. Cough A.L., Gallant R.B., 1976. Acalculous Gallbladder disease: a prospective study *Br. J. Surg.*; **63:** 970–978.
3. Toouli J. Roberts Thomson I.C., Dent J, et al. 1985; Manometric disorders in patients with suspected sphincter of oddi dysfunction. *Gastroenterology* **88:** 1243–1250.
4. Gunn A, Keddie N.C., Fox H. 1973; Acalculous gallbladder disease *Br. J. Surg.* **60:** 213–215.
5. Moody F.G., Calabuig R., Vecchio R., Ronkel N. 1990; Stenosis of sphincter of *oddi. Surg. Clin. North. Amer.* **70:** 1341–1354.
6. Stephens RV, Burdick G.F. 1986; Microscopic transduodenal sphincteroplasty and transampullary septoplasty for papillary stenosis. *And. Surg.* **152:** 621–627.
7. Carter R, Thomson R.J., Brennan O, Hinshaw D.B. 1963; Volvulus of the gallbladder S.G.O. **116:** 105–108.
8. May R.E. 1967; An unusual case of torsion of the gallbladder *Br. J. of Clin.* Practice **21:** 191–193.
9. Reid D.R.K., Rogers I.M. 1975; The negative cholecystogram in gallbladder disease. *Br. J. Surg.* **62:** 581–583.
10. Gilliant T.M. Traverso L.W. 1990; Acalculous gallbladder. *Am J. Surg.* **159:** 489–492.
11. Maingot R. 1961; Anomalies of the gallbladder and bile ducts: benign strictures and postoperative strictures of the bile ducts in Maingot abdominal operations. Fifth edition Vol.1 pp. 800–810.
12. Kozukz S, Tsubone M, Hachisuka K. 1982; Relation of adenoma to carcinoma in the gallbladder Cancer, **50:** 2226 –2234.

